# Localization of the First Mandibular Molar Roots in Relationship to the Mandibular Canal in Small Breed Dogs—A Tomography Imaging Study

**DOI:** 10.3389/fvets.2021.684763

**Published:** 2021-05-12

**Authors:** Han Chia, Kendall Taney, Don Hoover, James B. Robertson, Lenin A. Villamizar-Martinez

**Affiliations:** ^1^Center for Veterinary Dentistry and Oral Surgery, Gaithersburg, MD, United States; ^2^Veterinary Dental Clinic of North Carolina, Durham, NC, United States; ^3^Office of Research, College of Veterinary Medicine, North Carolina State University, Raleigh, NC, United States; ^4^Dentistry and Oral Surgery Service, Department of Clinical Science, North Carolina State University, Raleigh, NC, United States

**Keywords:** molar tooth, mandibular canal, tooth root, cone-beam computed tomography, high-definition computed tomography, small breed dogs

## Abstract

The intimate relationship between the mandibular canal (MC) and the first mandibular molar tooth presents challenges when performing dentoalveolar surgical procedures due to the probability of causing iatrogenic injury to the inferior alveolar neurovascular bundle. Superimposition between the MC and the first molar (M1) tooth roots is often observed on intraoral dental radiographs in small breed dogs. However, due to the radiograph's bidimensional nature, it is impossible to determine the buccal or lingual localization of the first molar roots with respect to the MC. Thus, this study's objective was to determine the localization of the first molar tooth's roots in relation to the position of the MC and their overlapping percentage with the canal in small-bodyweight dogs (<15 kg) using tomographic diagnostic images. For this, cone-beam computed tomography and high-definition computed tomography exams from 103 small breed dogs (under 15 kg) were retrospectively assessed to determine the lingual or buccal localization of the first molar tooth's roots with respect to the MC and the degree of overlap of the roots with the canal. In conclusion, most of the roots of M1 of dogs under 15 kg were located at the MC's lingual aspect (82.7%) with an overall superimposition median with the MC of 100 and 90% for the mesial and distal roots, respectively. Straddle tooth roots were not a common anatomical presentation in the dogs of this study.

## Introduction

The mandibular canal (MC) is a hollow space that carries the inferior alveolar neurovascular bundle, which innervates and provides blood supply to the gingiva, teeth, and rostral soft tissue of the mandible (1, 2). In the dog, the MC begins at the mandibular foramen located at the ventral region of the temporalis muscle insertion on the medial aspect of the ramus of the mandible. The inferior alveolar neurovascular bundle runs rostrally through the MC from the mandibular foramen to its end at the distal, middle, and rostral mental foramina on the buccal surface of the mandible at the level of the second premolar and canine teeth (1, 3). Knowledge of the MC's position regarding the mandibular teeth is of paramount importance since iatrogenic trauma of the inferior alveolar neurovascular bundle during dentoalveolar surgical procedures has been associated with intraoperative hemorrhage and temporary or permanent postoperative paresthesia or pain (4, 5).

The mandibular first molar (M1) is the largest two-rooted tooth of the mandible in domestic canines, and portions of the roots are adjacent to the MC. The cusps contain both a sharp edge and a flat edge for its function. The mesial end is sharp and intended for shearing, whereas the distal end is flat and used for grinding (6). Due to this tooth's size, it is common to see the M1 tooth roots and MC overlapping on the intra-oral dental radiographs. A previous intraoral radiographic study evaluated the relationship between patient body weight and the M1 size. These authors showed that dogs under 10 kg presented larger M1s than the mandibular height, with the M1 tooth roots extending ventrally to the MC (7).

Further computed tomography (CT) studies, performed in mesaticephalic and brachycephalic dogs of different sizes, demonstrated the dorsal positioning of the roots of M1 in reference to the position of the MC. Although it was suggested that small brachycephalic dogs might have a lingual or buccal positioning of the roots of M1 in reference to the MC, the lack of small breed specimens used in these studies could not confirm this assumption (8, 9). More recently, a cone-beam CT (CBCT) study performed in mesaticephalic canine cadaver heads of different sizes showed that 66% of the assessed M1 roots presented some degree of superimposition with the MC, with 73.3% of lingual roots within cortical bone of the mandible. This study also suggested that small breed dogs may have a higher incidence of more than 50% of superimposition between the M1 tooth roots and the MC (10).

Although intraoral radiographs are widely used to assess the dentoalveolar complex in veterinary patients, distortion and superimposition of dental structures and surrounding tissues are associated with this diagnostic imaging technique's bidimensional nature. CT and CBCT have been used in human and veterinary dentistry to evaluate the maxillofacial and dentoalveolar structures, where intraoral radiography has proven insufficient (10–14). Tomography imaging multiplanar reconstruction (MPR) and 3-D rendering provide the clinician superior visualization of anatomical structures and pathology without the superimposition of surrounded structures (10, 12–16).

While some tomographic studies have shown the positioning of the M1 tooth roots with the MC in different skull sizes and conformations in cadaver dogs, to the best of our knowledge, the relationship between the MC and the M1 tooth roots has not been clarified in small breed dogs. Thus, this research aimed to establish the lingual vs. buccal localization of the M1 roots in reference to location of the MC and determine the superimposition percentage between the M1 roots and the MC in small breed dogs (<15 kg), using tomographic images. We hypothesize that M1 roots are most likely to be located on the MC's lingual side with a high likelihood of 100% superimposition with the MC.

## Materials and Methods

Diagnostic imaging records from 103 client-owned small-size adult dogs (<15 kg), who underwent CBCT or high-definition CT (HDCT) exams for dentoalveolar or maxillofacial structures evaluation at two veterinary dentistry and oral surgery facilities, were retrospectively evaluated. The demographic data collected from the medical record for each patient included weight, breed, and gender. Exclusion criteria included missing one of the M1 teeth, advanced periodontitis, mandibular fracture, malformation, or neoplastic process at the M1 region. This study did not involve the use of animals, and therefore, ethical approval was not necessarily required. Power calculations were performed to determine that a sample size of 100 specimens was needed for 90% power, assuming a 5% significance level.

All tomographic exams were performed with the patients under general anesthesia. Anesthetic protocols were determined by a board-certified anesthesiologist or primary surgeon for each patient according to their clinical status. Physical oral examination and blood panels (complete blood count and chemistry with electrolytes) were obtained before anesthesia induction. For each patient, the head was scanned with the long axis of the body of the mandibles parallel to the headstand using a CBCT mobile unit^1^ at 0.3-mm voxel size, 24 × 14 cm field of view, 120 kVp, 57.6 mAs, and 20 s; and an HDCT scanner^2^ at 0.15 mm voxel size, 16 × 16 cm field of view, 70 kVp, 70 mAs, and 7 s. Multiplanar reformation (MPR) using suitable bony window and level settings was performed with a free DICOM viewer software for imaging evaluation.^3^ The images were reviewed by a board-certified veterinary dentist.

### Ligual vs. Buccal Localization of the Roots

**Figure 1:**
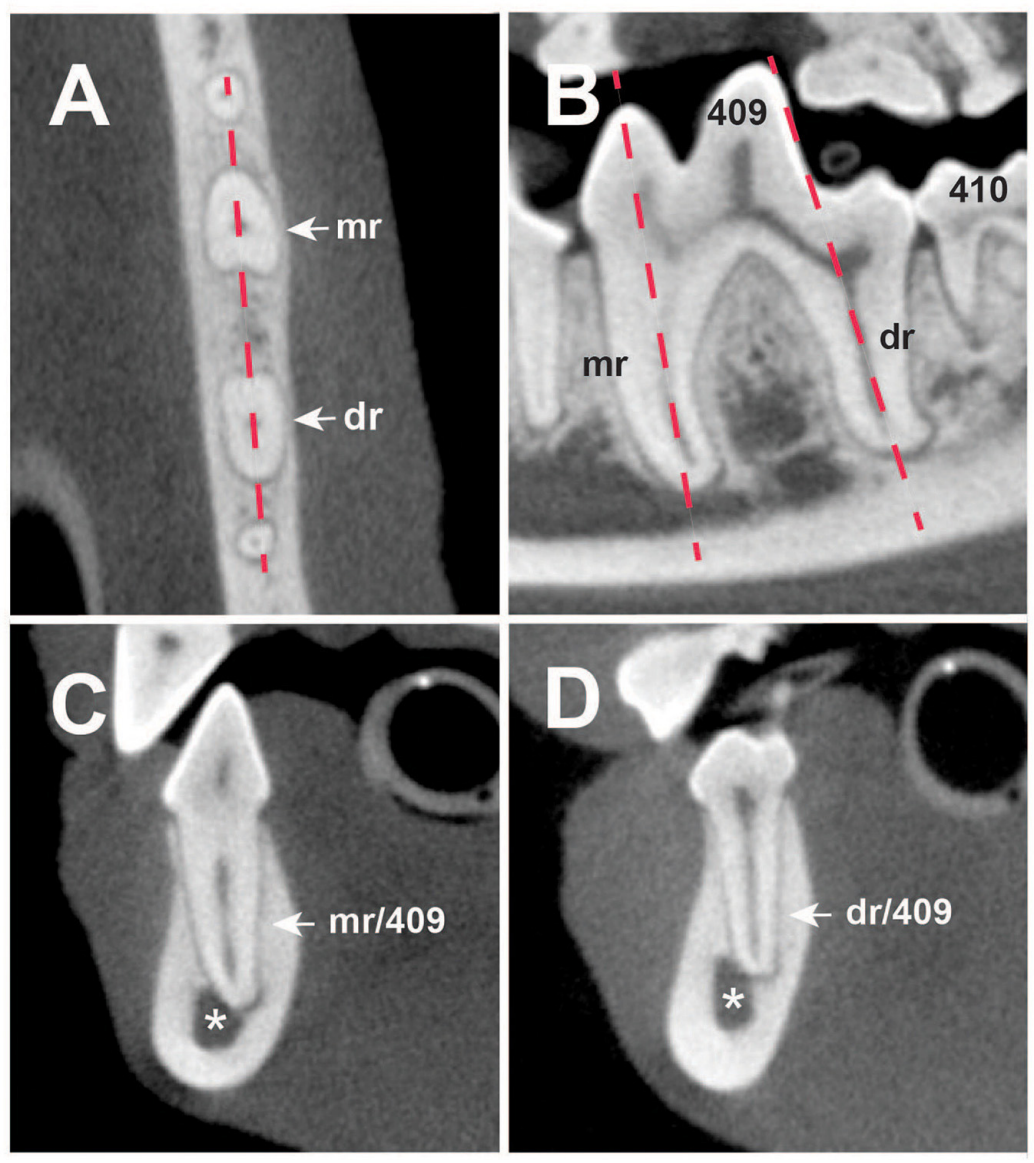
**(A)** Dorsal, **(B)** sagittal, and **(C,D)** transversal multiplanar cone-beam computed tomography (CBCT) reconstructions of the right mandible of a 4-year-old English Cocker Spaniel. **(A)** Dashed line shows the sagittal reconstruction of 409 displayed on **(B)**. **(B)** Dotted lines present the axis of the mesial (mr) and distal (dr) roots of 409. **(C)** Mesial root transversal reconstruction (mr/409). Distal root transversal reconstruction (dr/409). (Asterisk) Mandibular canal; (409 and 410) right mandibular first and second molar teeth.

Dorsal and sagittal MPRs of each mandibular body in each patient allowed localization of the M1 mesial and distal roots. The sagittal MPR of M1 was set by lining up the root canals of both roots, while transversal MPRs were set at each root's axis (Figure 1). On the transversal MPRs, the roots' location with the MC was categorized and recorded for statistical comparisons.

**Figure 2:**
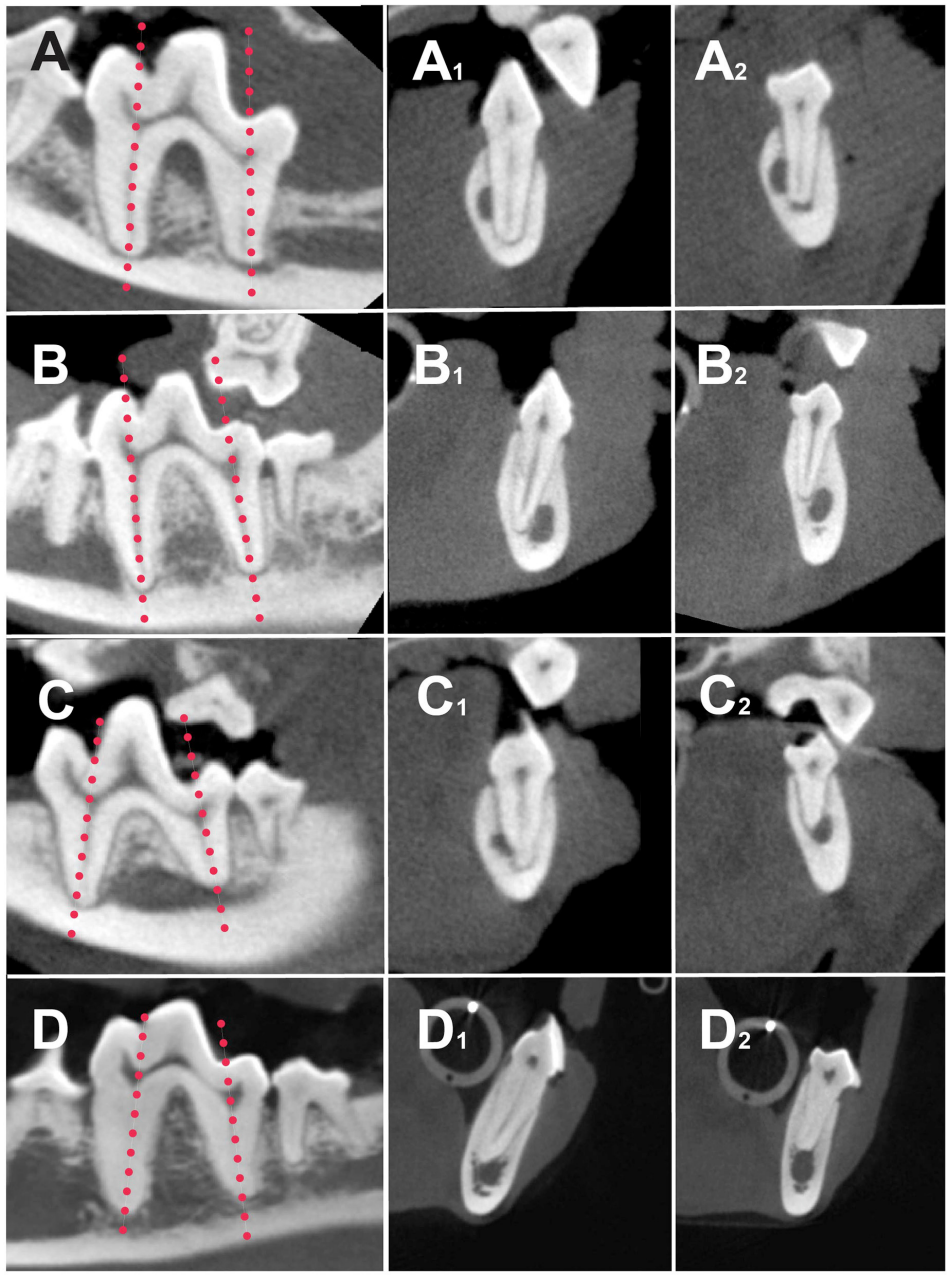
Sagittal **(A–D)** and transversal high-definition computed tomography (HDCT) reconstructions of the mesial **(A**_**1**_**-D**_**1**_**)** and distal roots **(A**_**2**_**-D**_**2**_**)** of different dogs showing different root localization. Dotted lines on **(A–D)** show the axis of the roots for the transversal reconstructions. **(A**_**1**_**,A**_**2**_**)** Both mesial and distal roots are located on buccal side of the mandibular canal (MC). **(B**_**1**_**,B**_**2**_**)** Both mesial and buccal roots are located on lingual side of MC. **(C**_**1**_**,C**_**2**_**)** Straddle tooth. **(C**_**1**_**)** Mesial root buccal to the MC; **(C**_**2**_**)** distal root lingual to the MC; **(D**_**1**_**)** mesial root lingually located; **(D**_**2**_**)** distal root dorsally located.

The buccal or lingual position of the mesial and distal roots was analyzed individually. Each tooth was also classified as having both roots in a buccal, lingual, or straddle position. Straddle teeth were defined as the mesial and distal roots located on opposite sides of the MC. Teeth with one or both roots dorsally located to the MC were defined as dorsal tooth root position and excluded from the superposition percentage of M1 tooth roots and MC (Figure 2). Tooth root localization for a single tooth and its contralateral equivalent were recorded for statistical purposes.

### Superimposition Between the Mandibular Canal and M1 Tooth Roots

**Figure 3:**
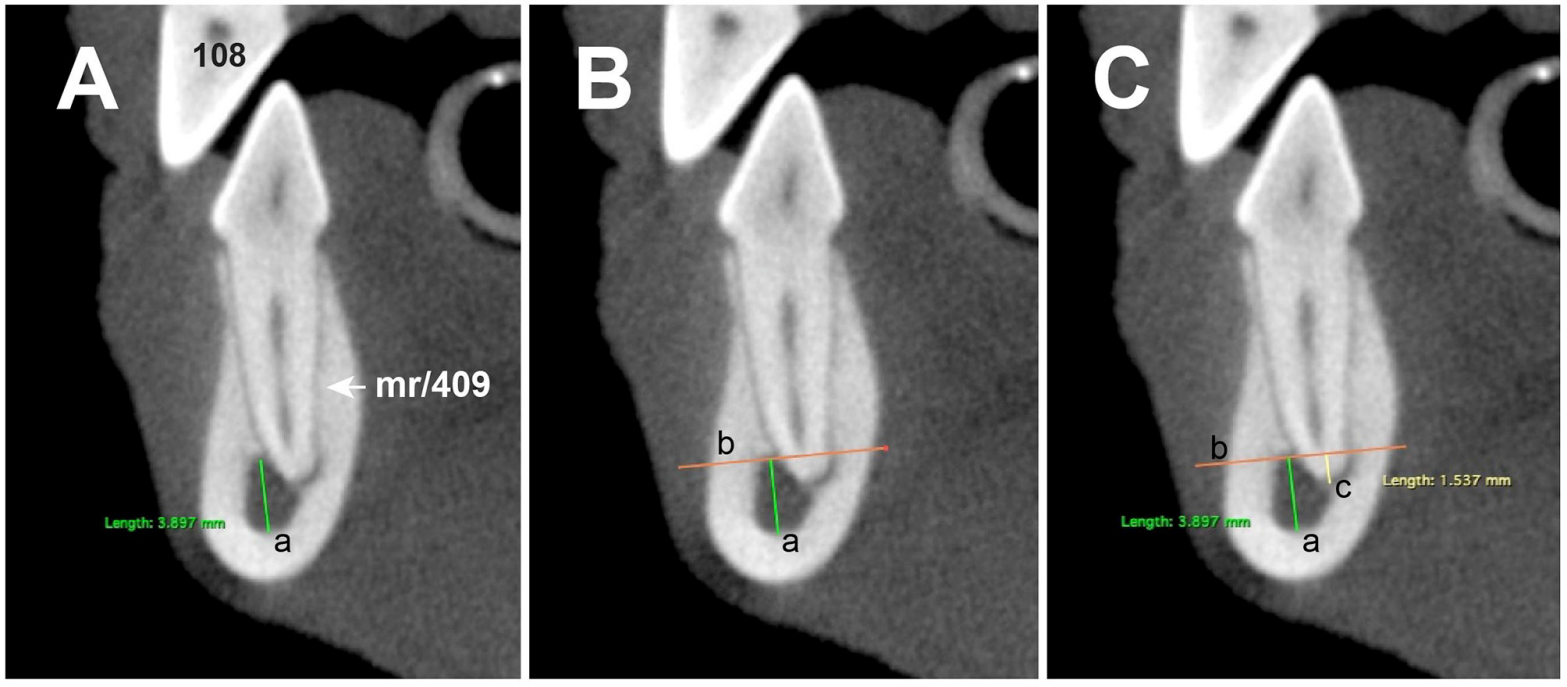
Transversal cone-beam computed tomography (CBCT) reconstructions showing how the superimposition percentage was obtained. **(A)** Line-a parallel to the axis of the M1 root is drawn to determine the diameter of the mandibular canal (MC). **(B)** Line-b is drawn perpendicular to line-a at the dorsal margin of the MC transecting the portion of the root that would appear superimposed with MC in a dental radiograph. **(C)** Line-c corresponds to amount of root superimposed.

The superimposition percentage between the MC and M1 tooth roots was calculated by comparing the MC height with the amount of superimposed M1 root along the same axis. The tooth root portion overlapping the MC was calculated on the transversal reconstruction. For this, each M1 root, the MC's vertical diameter, parallel to the tooth root axis, was measured. Second, at the MC's dorsal border, a horizontal line was drawn to form a right angle with the line used to measure the MC diameter. This line transected the portion of the root that was subsequently measured and compared with the MC's vertical diameter to determine the superimposition percentage (Figure 3).

### Statistical Analysis

All statistical analyses were conducted in R version 4.0.2^4^ using packages *lme4, lmerTest*, and *ggplot2*.^5^ Comparisons in rates of root locations were done via Z testing using the Normal approximation to the binomial. Comparisons of rates of root locations with continuous variables were done via mixed logistic regression with a random intercept for each patient. Comparisons of overlap were performed via mixed linear regression with weight and location of root as predictors with a random intercept for each patient; 95% confidence intervals were created using the Normal approximation to the Binomial distribution. Significance was defined as *p* < 0.05.

## Results

A total of 103 patients met the inclusion criteria proposed in this study. Twenty-four different breeds were presented within our sample population (Table 1). The most prevalent represented breeds were Dachshunds (*n* = 24), Chihuahuas (*n* = 14), and Yorkshire terriers (*n* = 10). Quantitative data were collected based on root location and superimposition percentage between the MC and the mesial and distal roots of M1.

**Table 1:** Breed distribution.

**Breed**	***n***	**Breed**	***n***
Beagle	1	Miniature Dachshund	1
Bichon Frise	6	Miniature Schnauzer	1
Border Terrier	3	Pomeranian	2
Boston Terrier	1	Poodle	8
Cavalier King Charles Spaniel	4	Miniature Poodle	1
Chihuahua	14	Pug	1
Dachshund	24	Rat Terrier	1
English Cocker Spaniel	1	Shetland Sheepdog	1
Havanese	3	Shih Tzu	8
Jack Russell terrier	3	Staffordshire Bull Terrier	1
Maltese	3	West Highland Terrier	3
Miniature Pinscher	2	Yorkshire Terrier	10

### Localization of Roots

Of all roots evaluated, there were 341 (82.7%) roots located at the lingual aspect of the MC (lingual roots), 59 (14.3%) roots located at the buccal aspect (buccal roots), and 12 (3.0%) roots located dorsal to the MC (dorsal roots). The teeth presenting tooth roots dorsally located to the MC were excluded for the remaining study. There was a total of 16 (8.2%) straddle teeth, 21 (10.8%) teeth with both roots on the buccal side, and 158 (81%) teeth with both roots on the lingual side of the MC.

When comparing the mesial and distal roots' localization, both roots were predominately located at the MC's lingual aspect (*p* = 0.001). The probability of buccal tooth roots, with 95% confidence, was between 13.3 and 16.2%, and the rate of lingual roots was between 83.8 and 86.7%. Table 2 summarizes the position of the mesial and distal roots regarding the MC. Symmetrical M1 root positioning was defined as having both M1s roots in both mandibles on the same side of the MC. A significant statistical difference was observed when comparing the localization of the M1 roots between both mandibles, where 81 dogs (87.1%) presented with symmetrical tooth root localization (*p* < 0.001). With 95% confidence, the symmetry and asymmetry rate were between 84.2–90% and 10–15.8%, respectively.

**Table 2:** M1 tooth root localization.

	**Root position**		
**Roots**	**Lingual**	**Buccal**	**Dorsal**
Mesial	165	59	7
Distal	176	25	5
Total (%)	341 (82.7%)	59 (14.3%)	12 (2.9%)

When one straddle tooth was present, the probability with 95% of confidence of having a second straddled M1 tooth was between 8.3 and 22.5%. Mandibles with no straddled M1s were more common than mandibles with straddled M1s (*p* = 0.001). With a 95% confidence, the probability of having straddled M1s was between 6.6 and 9.8%, while that for no straddled M1s was between 90.2 and 93.4%. Therefore, unilateral presentation of straddle M1s was the most common presentation in this study (*p* = 0.011).

Excluding Dachshunds, there was no association with sex, breed, or weight with root position or symmetricity in the dogs of this study. All Dachshunds were found to have only lingually located M1 tooth roots (*p* = 0.015), which indicated 100% symmetricity (*p* < 0.001). No Dachshunds were found to have straddle roots (*p* = 0.002).

### M1 Tooth Root/Mandibular Canal Superimposition

**Figure 4:**
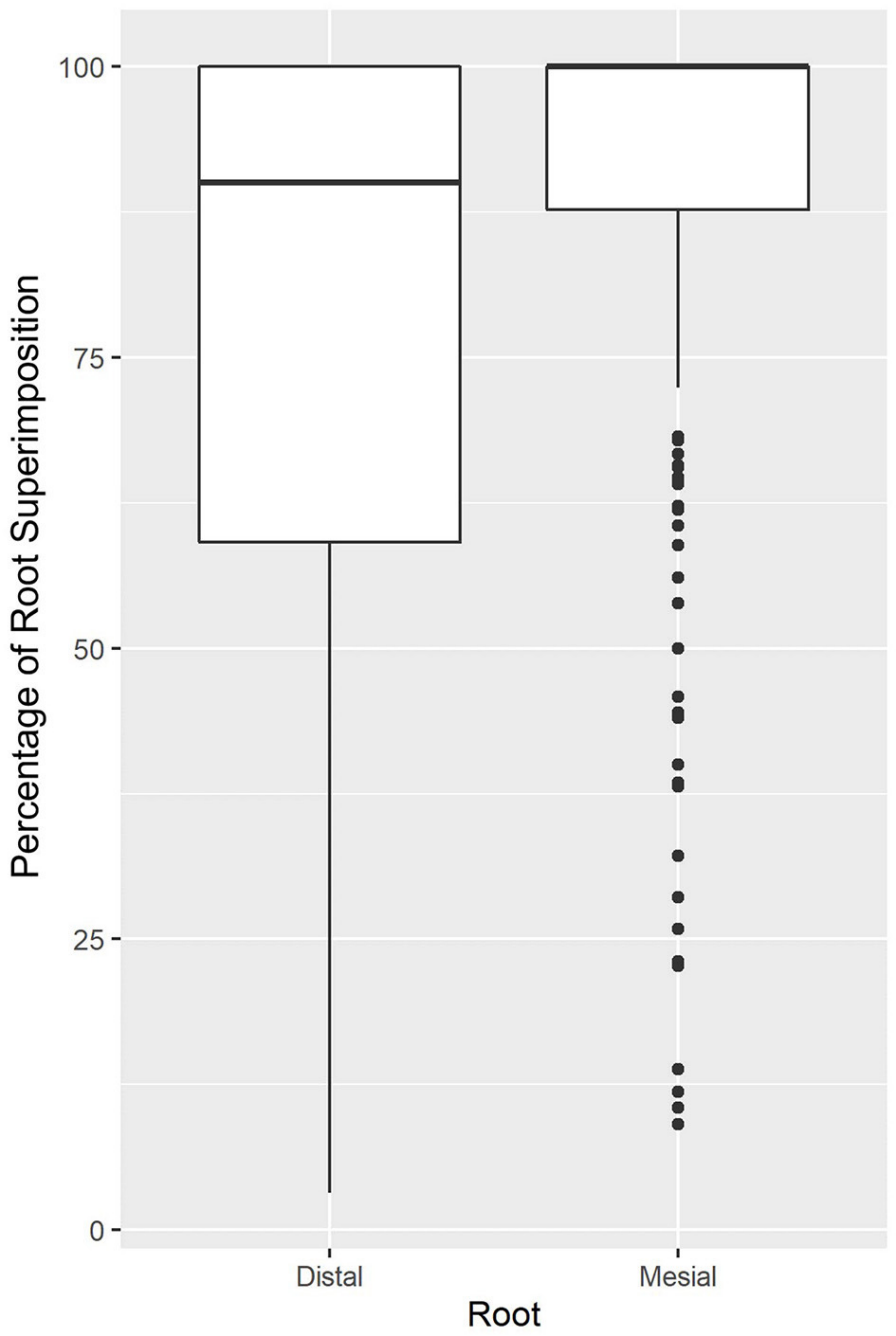
Superimposition percentage for the mesial and distal roots. The graph demonstrates that both distal and mesial roots of M1 showed a significant amount superimposed between the mandibular canal (MC) and M1, with superimposition medians of 90 and 100% for the distal and mesial, respectively.

Of all roots evaluated, 97% of M1 roots showed some level of superimposition with MC. From the 97% of roots, 56.3% showed 100% superimposition. The percentage of superimposition was significantly different between the mesial and distal roots. Full superimposition with the MC was presented by 136/202 (67.3%) mesial roots and 93/205 (45.4%) distal roots. Of all roots evaluated, mesial and distal roots showed superposition medians of 100 and 90%, respectively. The mesial and distal roots were 5.0 and 3.6 times more likely to be lingual if there was 100% superimposition of the M1 tooth roots and the MC. Figure 4 shows the distribution of superimposition between the mesial and distal roots.

**Figure 5:**
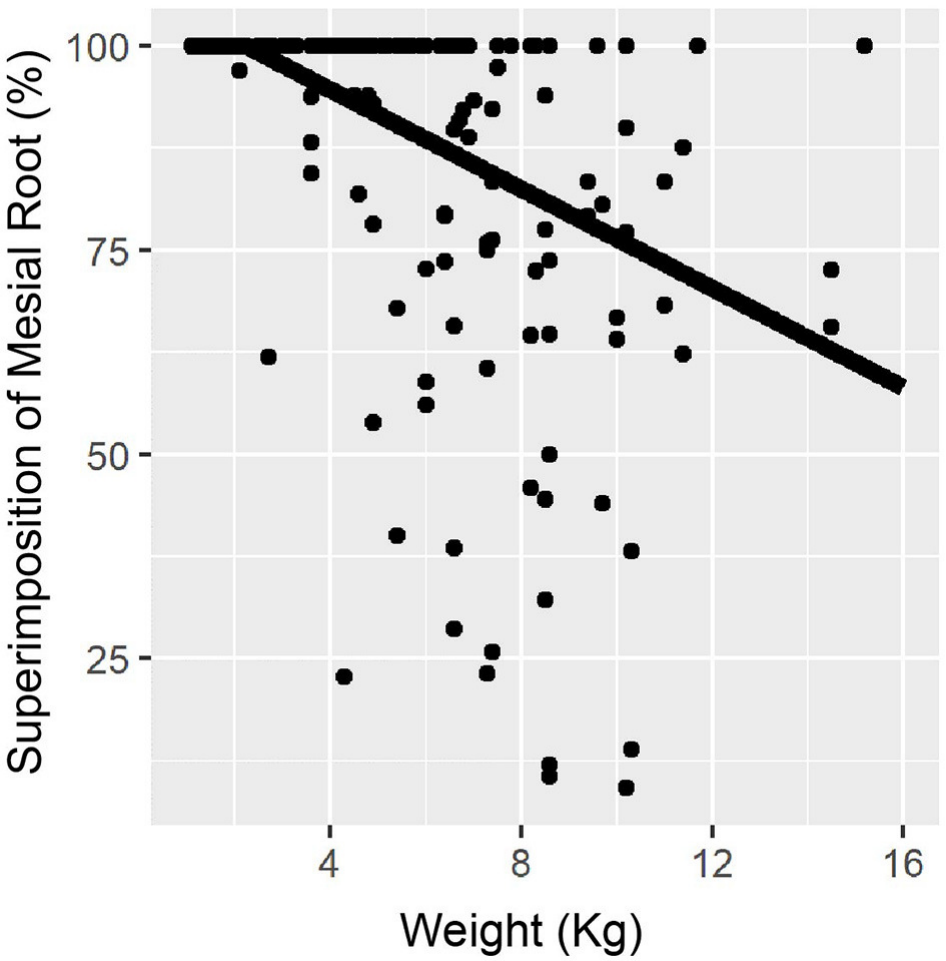
Relationship between mesial root superimposition and weight. Mesial root showed 100% of superimposition with the mandibular canal (MC) for patient <2 kg. Dogs heavier than 2 kg showed decreasing superimposition with the MC at a steady rate.

**Figure 6:**
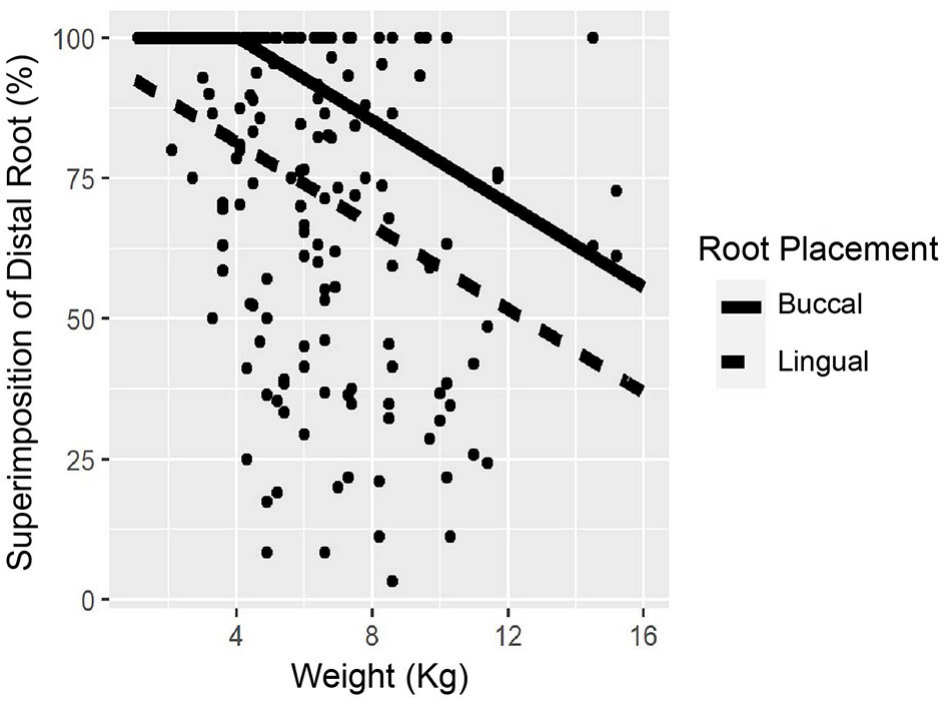
Relationship between distal root superimposition and weight. Distal root showed 100% superimposition with the mandibular canal (MC) for patients <4 kg. The lingual located root showed a consistent 18.75% less superimposition between the two structures.

When considering the weight and superimposition percentage with the MC, the mesial and distal roots were found to have a linear relationship. As the patient's body weight increased by 1 kg, the mesial roots decreased in superimposition average by 3.04% (*p* < 0.001), while the distal roots decreased 3.72% (*p* < 0.001) (Figures 5, 6). We found no relationship between straddle tooth roots and superimposition of the distal or mesial roots with the MC.

## Discussion

The first paper to describe the relationship between mandibular molar teeth and the MC was published in humans in 1986 based on intraoral dental radiographs (17). Since then, numerous published studies have elucidated the relationship between the molar teeth and the MC in different human populations using two- and three-dimensional diagnostic imaging techniques. In the veterinary field, this relationship has been evaluated with intra-oral radiographs, CT, and more recently CBCT (7–10). However, to the authors' knowledge, no studies have been performed using tomographic diagnostic images of small breed dogs. In this study, we investigated the relationship between the M1 roots and the MC in small canine patients (weight <15 kg) via tomographic images (CBCT and HDCT).

Our research reported that 82.7% of M1 roots were located at the MC's lingual aspect, a larger percentage compared with a previous study that showed a 73.3% of lingual localization of the roots. The difference between both studies may be related to the fact that the previous study likely enrolled more cadaver dog heads of larger sizes, which resulted in a high number of M1 roots located at the MC's dorsal aspect (10). Although we expected to find a lingual localization of the M1 roots as previously reported, enrolling only imaging records of small dogs increased the probability of superimposition between the roots and the canal and therefore the lingual pattern of the roots.

Our study demonstrated with 95% confidence that it is most common to find both M1 roots symmetrically located at the same side of the MC on both mandibles. However, the possibility of asymmetrical presentation of the roots (10–15.8%) must be recalled during dentoalveolar surgical procedures performed bilaterally in the mandibles. The use of bidimensional diagnostic imaging techniques such as intraoral radiographs is insufficient for evaluating the three-dimensional disposition of the roots and their location in reference to the canal. Tomographic images obtained with CT scans, CBCT, or HDCT are better diagnostic tools when evaluating the buccolingual relationship of the roots regarding the MC.

While tomographic investigations performed in dogs of different skull conformation demonstrated the MC position within the mandible and suggested the presence of straddle roots in small breed dogs, this assumption was not proven because of the small number of small breed dogs evaluated (8–10). However, straddle M1 teeth were not frequently observed in our study, and the probability of finding a contralateral straddle M1 was low (8.3 to 22.5%). Nevertheless, veterinary practitioners must be aware of this anatomical presentation during dental procedures. Straddle tooth roots may increase the predisposition to iatrogenic trauma of the inferior alveolar neurovascular bundle during dentoalveolar surgical procedures.

When comparing the root positioning by breed, Dachshunds were the only single breed with sufficient subjects (*n* = 24) for statistical analysis. Our study showed that both roots were symmetrically located in both mandibles on the MC's lingual region. The symmetry and the absence of straddle teeth in the Dachshunds could be associated with the skull confirmation of this specific breed. However, this assumption must be proven in further studies, including larger populations of dolichocephalic and mesaticephalic dogs.

In our study, 97% of roots showed some overlap with the MC, which was consistent with previous studies of the high probability of superimposition (7, 10).

In accordance with Gioso et al. (7), the superimposition percentage between M1 roots and the MC in our study was significantly associated with the patient's body weight, with superposition medians for the mesial and distal roots of 100 and 90%, respectively. In contrast to a similar study where only 21.2% of the roots evaluated presented 100% of superimposition with the canal, our study demonstrated that from all roots evaluated, 56.3% showed full superimposition (10). In line with previous literature, the highest superimposition percentages displayed in our research were associated with the fact that the roots of M1 in small breed dogs occupy a large volume of the mandible.

In accordance with Berning et al. (10), there were differences in superimposition percentage between the mesial and distal roots and the MC. Our study's data reflected a more considerable ventral extension of the mesial root of M1 into the mandibular body. In the clinical setting, the ventral extension of the dental roots affected by severe periodontitis has been associated with the high prevalence of pathological mandibular fracture, as it was previously suggested (18).

Superimposition between the MC and dental roots implies a formidable challenge for the veterinary practitioner. Dental extractions, retrieving retained roots, and surgical endodontic procedures could require extensive buccal alveolectomy, which would increase the probability of iatrogenic damage of the neurovascular tissue, thus causing intraoperative hemorrhage and temporary or permanent postoperative paresthesia or pain (4, 5). Lingual or buccal placement of dental elevators may cause severe trauma to the inferior alveolar neurovascular bundle since 97% of roots are located either lingual or buccal to the MC. Our study's results support the recommendations of previous studies regarding the mesial or distal placement of the dental elevator to decrease the possibility of iatrogenic trauma (8).

A lingual approach has been described in the literature for the extraction of the mandibular canine tooth to avoid the middle mental neurovascular bundle (19); however, no studies have been reported regarding the employment of this technique for other teeth of the mandible. To the authors' knowledge, there is no literature describing a lingual approach for M1 extraction. Additional research is needed to determine the safety and efficacy of a lingual approach for the M1 roots.

We reviewed the imaging records from two tomographic devices used in two different veterinary dentistry and oral surgery facilities. While our study was not designed to compare both machines, the DICOM images from both types of equipment allowed us to perform the necessary MPR for the assessment proposed in this study. In contrast, while intraoral radiography has proved to be a suitable diagnostic imaging technique for determining the superimposition between the MC and the dental roots (7), its bidimensional nature does not allow the veterinary practitioner to establish the roots' lingual or buccal location in a clinical setting.

A limitation of this study was the small number of dogs represented by specific breeds. Although the general population used in this research was sufficient to evaluate the data with 95% confidence, the Dachshund was the only breed that allowed us to investigate the incidence in a single breed with confidence. The lingual tooth root symmetry displayed by the Dachshund may be related to this dolichocephalic breed's anatomy. However, this assumption must be validated. In addition, novel studies are necessary to elucidate the MC's position in larger populations of small brachycephalic dogs.

In conclusion, most of the roots of M1 of dogs under 15 kg were located at the MC's lingual aspect (82.7%) with an overall superimposition median with the MC of 100 and 90% for the mesial and distal roots, respectively. Straddle tooth roots were not a common anatomical presentation in the dogs of this study.

## Data Availability Statement

The raw data supporting the conclusions of this article will be made available by the authors, without undue reservation.

## Author Contributions

HC: study concept, design, provided study material/cases, manuscript writing, and data analysis. KT: review of manuscript for important intellectual input. JR: data analysis, interpretation, and graphic presentation. DH: provided study material/cases. LV-M: study concept, design, provided study material or cases, manuscript writing, data analysis, review of manuscript for important intellectual input, and graphic presentation. All authors contributed to the article and approved the submitted version.

## Conflict of Interest

The authors declare that the research was conducted in the absence of any commercial or financial relationships that could be construed as a potential conflict of interest.

## Footnotes

^1^VetCAT™, Xoran Technologies, Ann Arbor, MI.

^2^Epica Vimago veterinary CT scanner, San Clemente, CA.

^3^Horos, version 3 (LGPL-3.0).

^4^R Core Team (2020). R: A language and environment for statistical computing. R Foundation for Statistical Computing, Vienna, Austria. Available online at: https://www.R-project.org/.

^5^H. Wickham. ggplot2: Elegant Graphics for Data Analysis. Springer-Verlag New York, 2016.
